# Mercurial Poisoning Amongst Hatters

**Published:** 1911-09-16

**Authors:** 


### Medico-Legal Points.

### MERCURIAL POISONING AMONGST HATTERS.

At Hyde County Court recently his Honour Judge Reginald Brown, K.C., gave his decision in a case under the Workmen's Compensation Act, in which the applicant was Herbert Burgess, a working hatter, and the respondents Messrs. Joseph Wilson and Sons, hat manufacturers, Denton. For fifteen years the applicant has been in the employ of the respondent's firm as a "proofer," at an average wage of £1 17s. 6d. a week. In the early part of last year he began to have symptoms of disease, which were subsequently certified by a doctor to be due to mercurial poisoning. Some of his teeth fell out, his gums were affected, and he could not retain food. It was contended that he contracted the disease while following his employment at the defendants' works, where he inhaled a vapour. It was admitted by the respondents that the applicant had mercurial poisoning, but they urged that they did not use mercury in manufacturing, and that it must have been in the fur when they bought the latter. In his judgment, his Honour asked whether a workman could be said to be employed in a process when the employer did not provide the mercury or even know it was there? His Honour thought not, and said it would open a very wide door if it were otherwise, and would establish liability wherever an employer used in his business any article procured elsewhere by him which, wholly or in part, was made of or contained or was even impregnated with lead, mercury, phosphorus, or arsenic. His Honour was of opinion that Schedule II., at any rate, of the Act only referred to a process in which the employer himself used the mercury in the ordinary acceptation of the term, and therefore that this case was not within the Act. He dismissed the application with costs, and invited an appeal.

